# Construction of a comprehensive predictive model for axillary lymph node metastasis in breast cancer: a retrospective study

**DOI:** 10.1186/s12885-023-11498-7

**Published:** 2023-10-24

**Authors:** Yan Li, Dong Han, Cong Shen, Xiaoyi Duan

**Affiliations:** PET/CT Center, The First Affiliated Hospital of Xi’an Jiaotong University, 277 Yanta West Road, Xi’an Shaanxi, 710061 China

**Keywords:** Breast cancer, Lymph node Metastasis, Radiomics, PET/CT, Ultrasound

## Abstract

**Purpose:**

The accurate assessment of axillary lymph node metastasis (LNM) in early-stage breast cancer (BC) is of great importance. This study aimed to construct an integrated model based on clinicopathology, ultrasound, PET/CT, and PET radiomics for predicting axillary LNM in early stage of BC.

**Materials and methods:**

124 BC patients who underwent 18 F-fluorodeoxyglucose (18 F-FDG) PET/CT and whose diagnosis were confirmed by surgical pathology were retrospectively analyzed and included in this study. Ultrasound, PET and clinicopathological features of all patients were analyzed, and PET radiomics features were extracted to establish an ultrasound model (clinicopathology and ultrasound; model 1), a PET model (clinicopathology, ultrasound, and PET; model 2), and a comprehensive model (clinicopathology, ultrasound, PET, and radiomics; model 3), and the diagnostic efficacy of each model was evaluated and compared.

**Results:**

The T stage, US_BIRADS, US_LNM, and PET_LNM in the positive axillary LNM group was significantly higher than that of in the negative LNM group (P = 0.013, P = 0.049, P < 0.001, P < 0.001, respectively). Radiomics score for predicting LNM (RS_LNM) for the negative LNM and positive LNM were statistically significant difference (-1.090 ± 0.448 vs. -0.693 ± 0.344, t = -4.720, P < 0.001), and the AUC was 0.767 (95% CI: 0.674–0.861). The ROC curves showed that model 3 outperformed model 1 for the sensitivity (model 3 vs. model 1, 82.86% vs. 48.57%), and outperformed model 2 for the specificity (model 3 vs. model 2, 82.02% vs. 68.54%) in the prediction of LNM. The AUC of mode 1, model 2 and model 3 was 0.687, 0.826 and 0.874, and the Delong test showed the AUC of model 3 was significantly higher than that of model 1 and model 2 (P < 0.05). Decision curve analysis showed that model 3 resulted in a higher degree of net benefit for all the patients than model 1 and model 2.

**Conclusion:**

The use of a comprehensive model based on clinicopathology, ultrasound, PET/CT, and PET radiomics can effectively improve the diagnostic efficacy of axillary LNM in BC. Trial registration: This study was registered at ClinicalTrials Gov (number NCT05826197) on 7th, May 2023.

## Introduction

BC is a commonly occurring primary malignant tumor in women with high heterogeneity and varying degrees of malignancy [1, 2]. Surgical intervention is essential for its early diagnosis and treatment. The status of axillary LNM is an important factor affecting the prognosis of BC patients [1, 3]. Currently, clinicians mainly rely on mammography, ultrasound, MRI [4] and PET/CT for the diagnosis of axillary LNM in BC [5]. However, the sensitivity or specificity are unsatisfactory [6]. Axillary lymph node biopsy is relatively accurate, but it is an invasive procedure that may cause complications such as lymphedema, pain, numbness, limitation of shoulder movement, and nerve injury [7]. So, a new noninvasive method for preoperative axillary lymph node assessment is needed. Several studies have demonstrated the potential role of radiomics in the staging, prognosis, and evaluation of BC [8]. Recent studies have shown that radiomics have a good predictive power for evaluating LNM various cancers [9, 10]. Therefore, the study of indirectly evaluating the metastatic status of axillary lymph nodes by extracting the characteristics of breast cancer nodes using radiomics has become a hot topic.

## Materials and methods

### Study participants

**Fig. 1:**
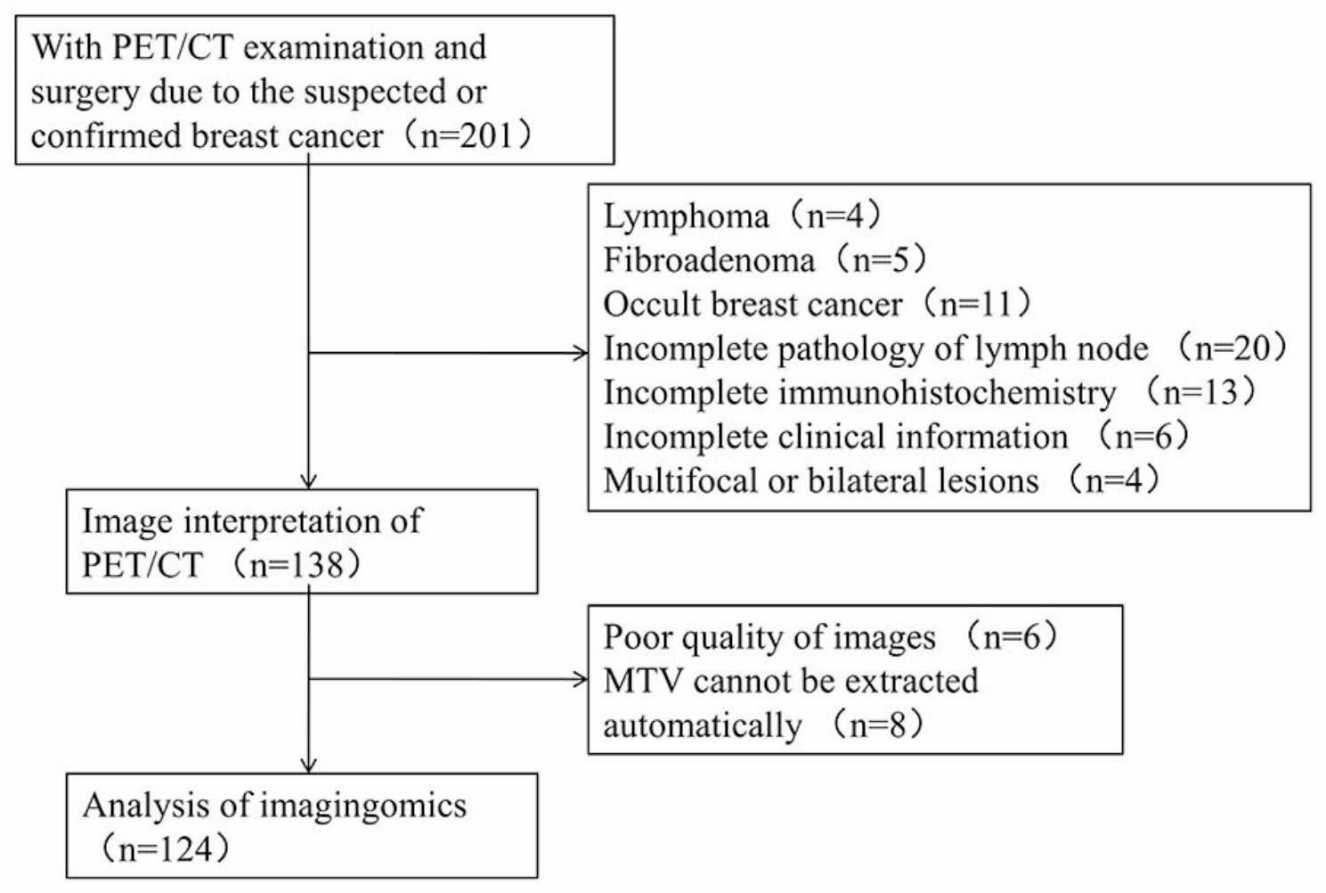
Study workflow

Patients with suspected BC undergoing PET/CT at our hospital, from November 2016 to April 2022, were selected via picture archiving and communication as well as hospital information systems, based on the inclusion process depicted in Fig. 1. This study was conducted in accordance with the principles of the Declaration of Helsinki and was reviewed and approved by the Medical Ethics Committee of the First Affiliated Hospital of Xi’an Jiaotong University(No. IRB-SOP-AF-16). All data were anonymized prior to analysis. Tumor staging was done in accordance with the eighth edition of the American Joint Committee on Cancer staging manual [11].This study was funded by the Department of Science and Technology of Shaanxi Province(No. 2023-YBSF-480), and registered with ClinicalTrials.gov (Date of first registration: 24/04/2023, ClinicalTrials.gov Identifier: NCT05826197).

Inclusion criteria: (1) ^18^ F-FDG PET/CT for breast lesions; (2) women with pathologically confirmed BC (age ≥ 18 years); (3) no history of surgery, radiotherapy, or chemotherapy before ^18^ F-FDG PET/CT; and (4) interval between ^18^ F-FDG PET/CT and puncture/surgery ≤ 2 weeks.

Exclusion criteria: (1) multifocal, bilateral, or occult BC; (2) incomplete clinical or pathological data; (3) poor PET/CT image quality preventing automated segmentation of metabolic tumor volume (MTV); and (4) concomitant malignant tumors.

### PET/CT imaging methods

PET/CT was performed on all patients using a 64-detector scanner (Gemini TF PET/CT, Philips, Netherlands). ^18^ F-FDG was synthesized by GE MINItrace mini cyclotron and Tracerlab FX-FDG synthesizer, and the synthetic precursor kit was purchased from ABX, Germany. The synthesized ^18^ F-FDG was released with a purity of ≥ 95%, and the quality was assured to be suitable for human injection. Patients fasted for at least 6 h before injection and had a fasting blood glucose level of less than 12.0 mmol/L. ^18^ F-FDG (dose 370 MBq/kg) was intravenously injected from the contralateral upper extremity of the affected mammary gland. The patients were encouraged to have sufficient water intake and rest for 60 min. The parameters for the CT scans were as follows: tube voltage 120 kV, tube current 300 mA, layer thickness 5 mm, layer spacing 5 mm, 512 × 512 matrix. PET collected 7–10 beds with 1.5 min/bed. PET images were corrected by the same machine used for CT data attenuation and reconstructed using an iterative method and time of flight. The imaging data were transferred to a workstation for image post-processing.

### Image interpretation

**Fig. 2:**
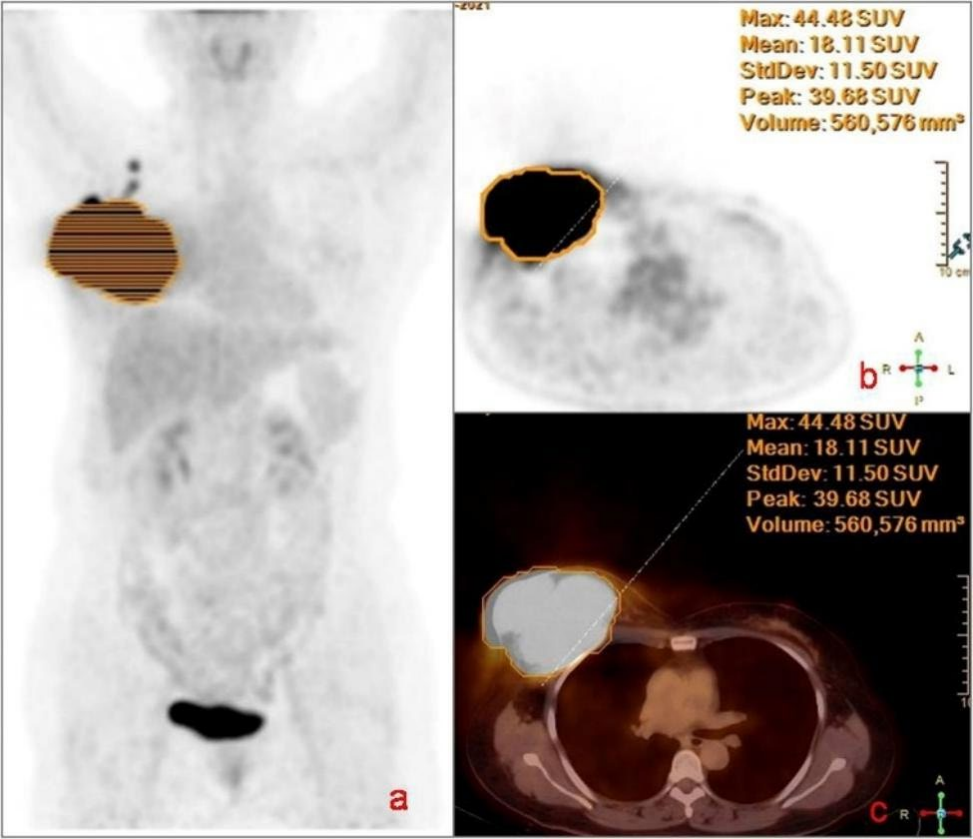
Metabolic parameters measurements of PET/CT scan. Female, 56 years-old, with BC. MIP image (**a**) showed the intense uptake of the BC lesion at the right side. The 3D VOI was delineated by physician with the rule of 40% of the maximum standardized uptake value (SUVmax) on the PET image (**b**) or PET/CT fusion images (**c**). SUVmax, SUVmean, SD, MTV were measured respectively

The PET/CT center’s chief physician and senior attending physician reviewed the images together and disagreement, if any, was resolved by consensus. The lesion was visually identified. A 3D region of interest (ROI) of the lesion was automatically outlined using the 40% threshold method, and PET metabolic parameters were measured, seen as Fig. 2.

Breast lesions with radionuclide concentrations greater than those in normal breast tissue were considered to be BC lesions, while lymph nodes with radionuclide concentrations greater than those in muscle tissue were considered to be metastatic lymph nodes.

### Radiomics

**Fig. 3:**
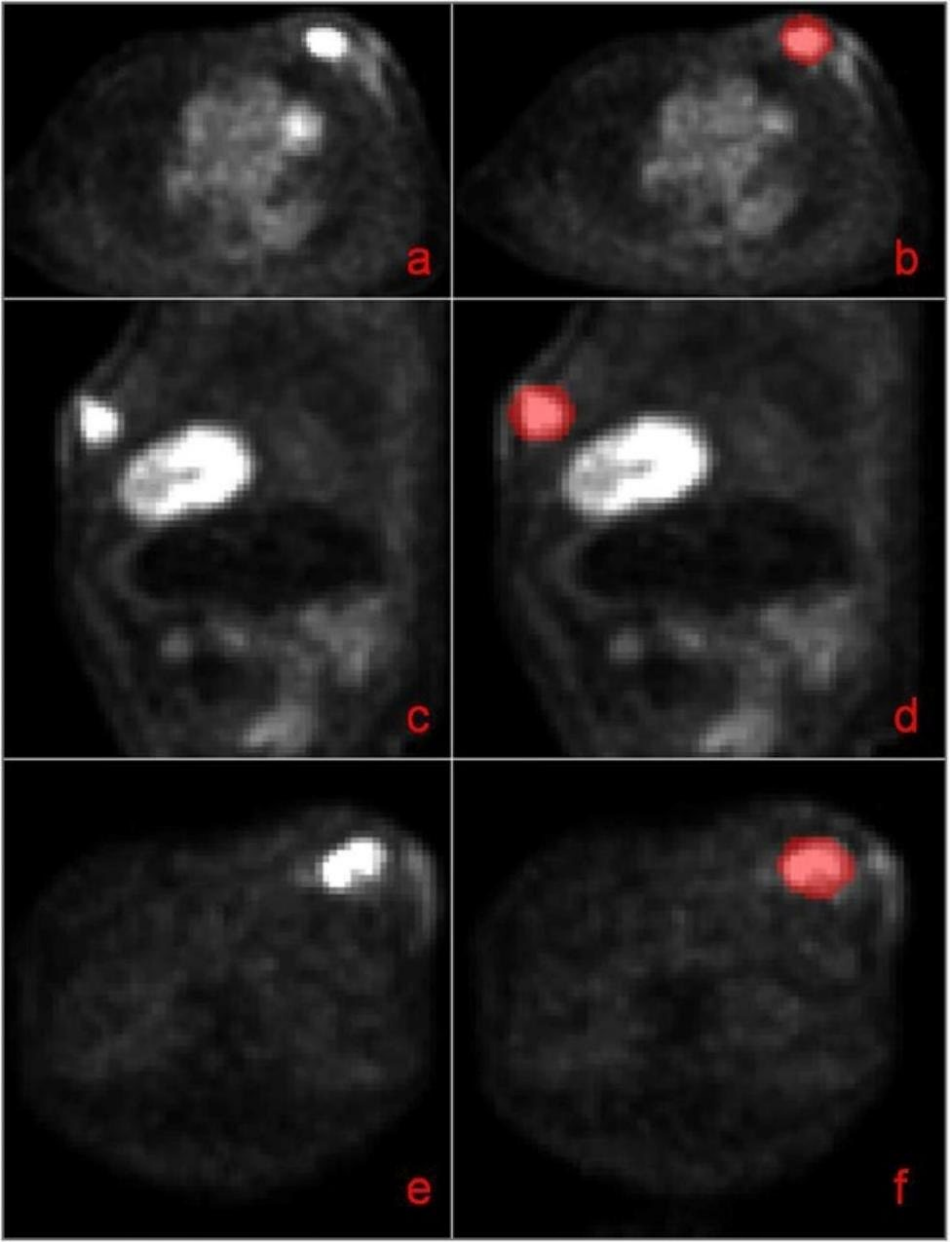
3D lesion segmentation. Axial (**a**), sagittal (**c**), and coronal PET images (**e**) of breast tumors, outlined axial (**b**), sagittal (**d**), and coronal images (**f**) of tumor ROIs

Image segmentation was performed using ITK-SNAP software [12] (version 3.6.0, http://www.itksnap.org/); Brush Style: circular, Brush Size: 10, Brush Options: 3D. The entire tumor volume was outlined on the PET image as ROI for segmentation, seen as Fig. 3. The lesions were marked by the attending physician and checked by the chief physician.

An open source Python package (PyRadiomics version 3.0.1 [13]) was used to extract the radiomics features from the ROI, and a total of 851 radiomics features were finally computed. These features were extracted and defined in accordance with the Image Biomarker Standardization Initiative.

### Clinical and pathological features

Breast imaging reporting and data system classification was used to classify all BCs involving lymph nodes. The histological grading of BC was assessed using the internationally accepted Nottingham tissue grading system [14]. BC specimens were fixed in 4% formaldehyde solution and embedded in paraffin wax, sectioned at a thickness of 4 μm. They were routinely stained with HE and then subjected to immunohistochemistry which included evaluation of estrogen receptor, progesterone receptor, human epidermal growth factor receptor 2, p53, and cell proliferation nuclear antigen Ki67.

### Statistical analysis

Statistical analysis was performed using R language (version 4.1.0. R Foundation for Statistical Computing, Vienna, Austria, URL https://www.R-project.org/) and SPSS® (version 25.0, IBM Corp, Armonk, NY, USA) software with a significance level of α = 0.05. The data obtained were expressed as mean ± standard deviation. Groups were compared using the independent samples t-test if they were normally distributed with equal variance, otherwise the Mann–Whitney U test was used for comparison. Categorical variables were compared using the χ^2^ test or Fisher’s exact test. The least absolute shrinkage and selection operator (LASSO) was used to downscale the radiomics features, and the Radiomics Score (RS) was established based on the coefficients of the downscaled features. Univariate and multivariate binary logistic regressions were used to construct three parametric models based on clinicopathology and ultrasound (model 1); clinicopathology, ultrasound, and PET (model 2); and clinicopathology, ultrasound, PET, and radiomics (model 3), for predicting LNM in BC. ROC curves were plotted and area under the curve (AUC) was calculated to evaluate the discrimination of the three models, and the AUCs of the three models were compared using the Delong test. Furthermore, 1000 bootstraps with put-back repeated sampling were used to internally validate the differentiation of the models and to calculate the corrected AUC. Calibration curves were plotted separately to evaluate the calibration of the three models. The net reclassification index (NRI) and integrated discrimination improvement index (IDII) were used to evaluate the inter-model improvement. Finally, decision curves were plotted to evaluate the net benefit of the three models for all patients.

## Results

### Comparison of general information

A total of 124 BC patients with a median age of 49 years-old (20–76 years) were included in this study, and the clinic-pathological characteristics of the axillary LNM negative group (n = 89) and the axillary LNM positive group (n = 35) were compared to identify potential diagnostic biomarkers of axillary LNM. The T stage, US_BIRADS, US_LNM, and PET_LNM in the positive axillary LNM group was significantly higher than that of in the negative LNM group (*P* = 0.013, *P* = 0.049, *P* < 0.001, *P* < 0.001, respectively), seen as Table 1. There were no statistical differences in age, tumor location, quadrant distribution, subtypes, grade, mol-subtypes, SUVmax, SUVmean, SD and MTV between the two groups (*P*>0.05), as shown in Table 1.

**Table 1:** Comparison of general information of patients in two groups

Parameters	LNM(-) (n = 89)	LNM(+) (n = 35)	*t/χ* ^*2*^	*p*
Age	48.33 ± 11.86	51.09 ± 10.62	-1.200	0.232
Tumor location			0.679	0.410
Left	48	16		
Right	41	19		
Quadrant distribution			7.488	0.187
Outer upper	35	13		
Outer lower	11	4		
Inner upper	27	9		
Inner lower	7	0		
Middle upper	3	4		
Middle lower	6	5		
T stage			12.617	0.013
Tis	3	0		
T1	52	10		
T2	26	19		
T3	6	3		
T4	2	3		
Subtypes			1.898	0.387
Invasive ductal carcinoma	77	33		
Invasive lobular carcinoma	9	2		
Ductal carcinoma in situ	3	0		
Grade			1.903	0.386
G1	3	0		
G2	39	13		
G3	47	22		
Mol-subtypes			4.402	0.221
LuminalA	23	6		
Luminal B	27	17		
HER2positive	25	6		
Triple-negative	14	6		
US_BIRADS			9.554	0.049
Bi-rads 1	1	1		
Bi-rads 3	2	1		
Bi-rads 4	55	11		
Bi-rads 5	18	12		
Bi-rads 6	13	10		
US_LNM			20.558	< 0.001
negative	79	18		
positive	10	17		
PET_LNM			32.065	< 0.001
negative	65	6		
positive	24	29		
SUVmax	7.91 ± 4.46	8.82 ± 5.46	-0.961	0.339
SUVmean	4.22 ± 2.39	4.32 ± 2.4	-0.195	0.846
SD	1.26 ± 0.86	1.44 ± 1.29	-0.909	0.365
MTV	31484.88 ± 153481.91	36169.74 ± 76683.17	-0.172	0.864

### LASSO regression, RS calculation

**Fig. 4:**
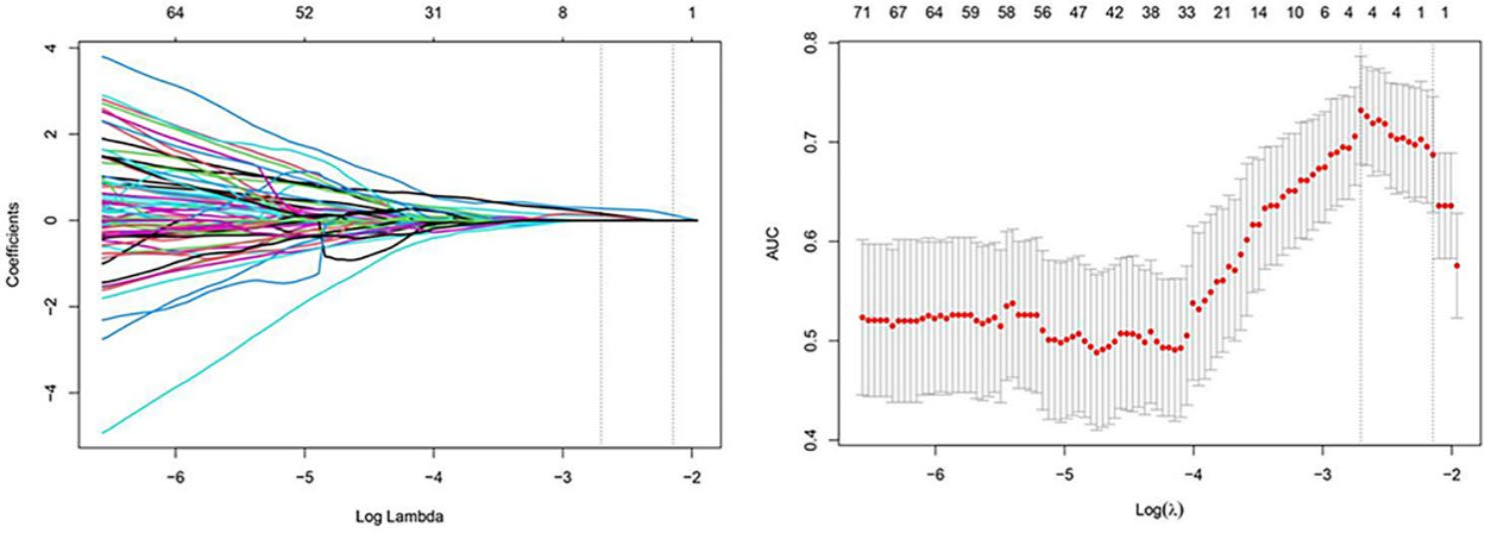
LASSO regression cross-validation diagram and regression coefficient diagram, the upper horizontal axis is the number of radiomics features corresponding to the models. The two vertical dashed lines in Fig. 4A show the two log (λ) values for minimum mean-squared error minimum and the increase of 1 SD (one standard deviation) mean-squared error minimum determined by cross-validation. Figure 4B shows that with the increase of log (λ), the radiomics features coefficients were gradually compressed to 0, and the number of features was reduced to 4 by the log (λ) with minimum mean-squared error minimum. LASSO, least absolute shrinkage and selection operator

**Fig. 5:**
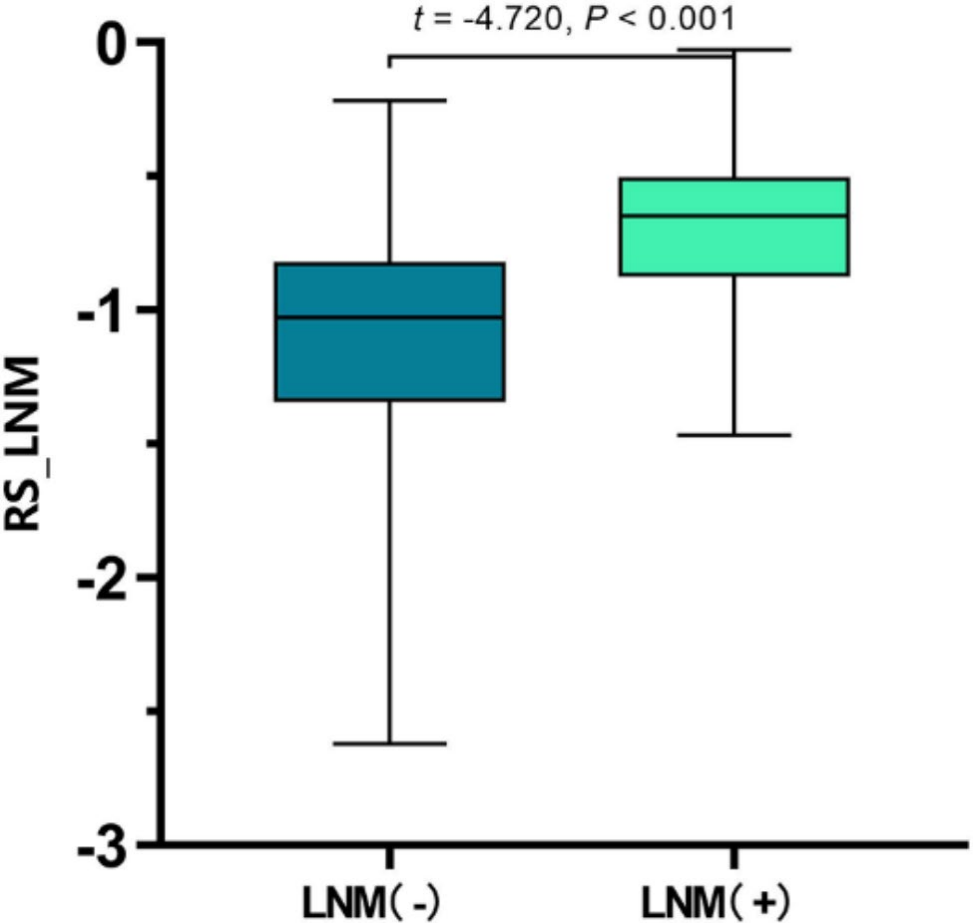
Comparison of RS_LNM between two groups

**Fig. 6:**
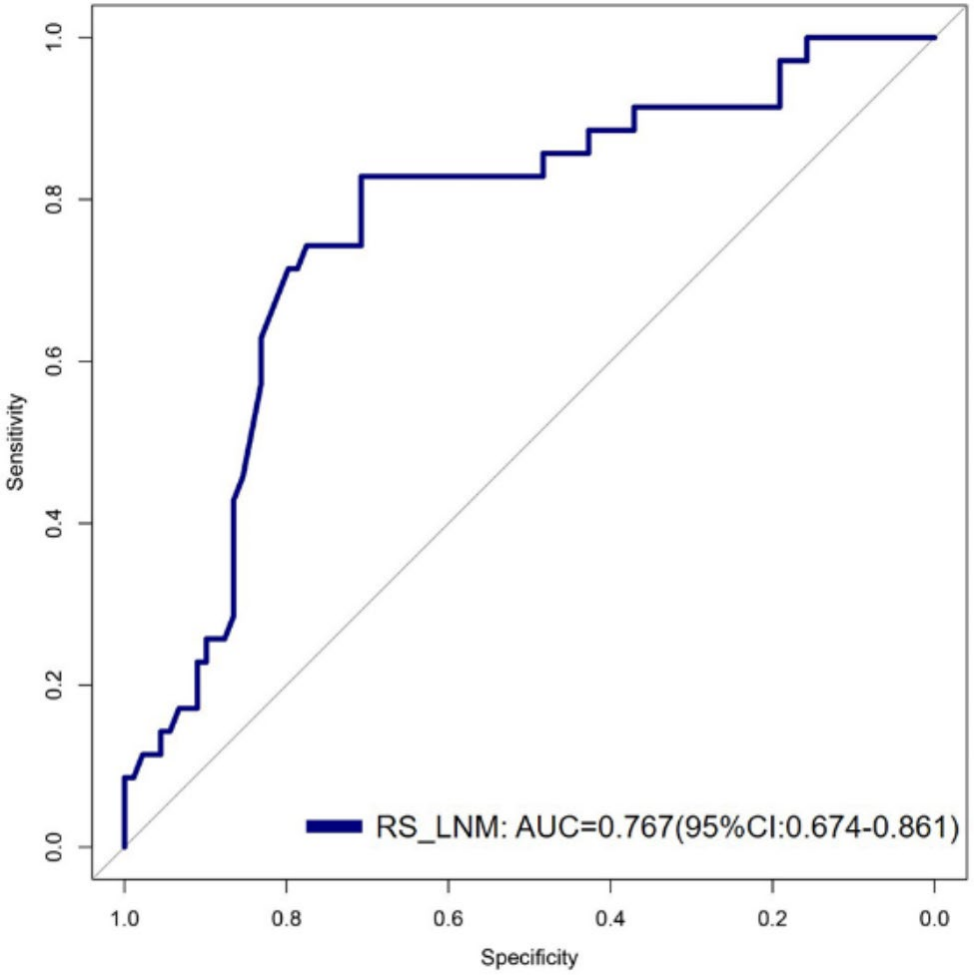
ROC curve analysis of RS_LNM

The radiomics features were normalized using Z-scoring and then downscaled using LASSO regression, and the optimal lnλ = -2.704 was determined by cross-validation, as shown in Fig. 4. Based on the linear weighting of the four radiomics features and their coefficients, radiomics score for predicting LNM(RS_LNM) was calculated, that was, Zoriginal_firstorder_10Percentile * 0.0891130 + Zoriginal_glszm_SizeZoneNonUniformityNormalized * 0.2768424 + ZwaveletLLH_firstorder_Skewness * 0.1603961 + ZwaveletHHH_glrlm_ ShortRunEmphasis * 0.1117953–0.9779143. RS_LNM for the group of negative LNM and the group of positive LNM were − 1.090 ± 0.448 and − 0.693 ± 0.344, respectively, with a statistically significant difference (*t* = -4.720, *P* < 0.001; Fig. 5). In the ROC analysis, the AUC was 0.767 (95% CI: 0.674–0.861; Fig. 6).

### Construction and comparison of the three prediction models

**Fig. 7:**
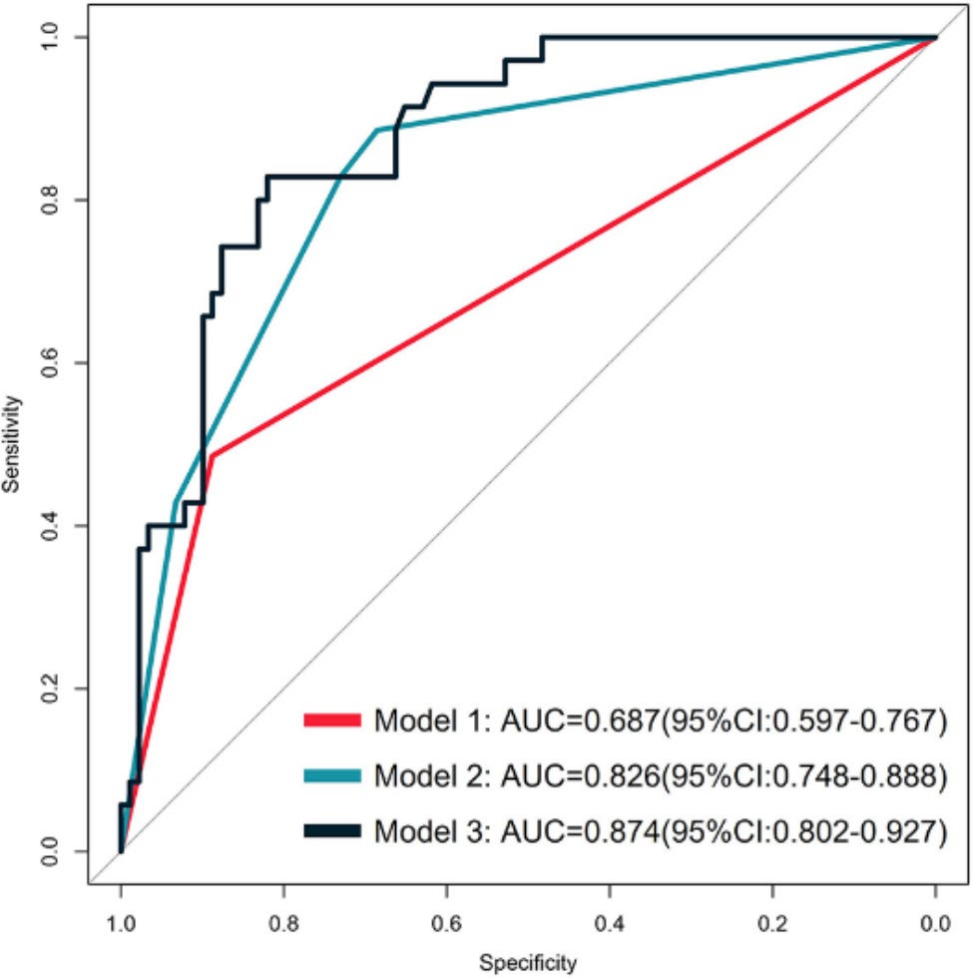
Comparison of ROC curves for the three models. Model 3 outperformed model 1 for the sensitivity (model 3 vs. model 1, 82.86% vs. 48.57%), and outperformed model 2 for the specificity (model 3 vs. model 2, 82.02% vs. 68.54%)

**Fig. 8:**
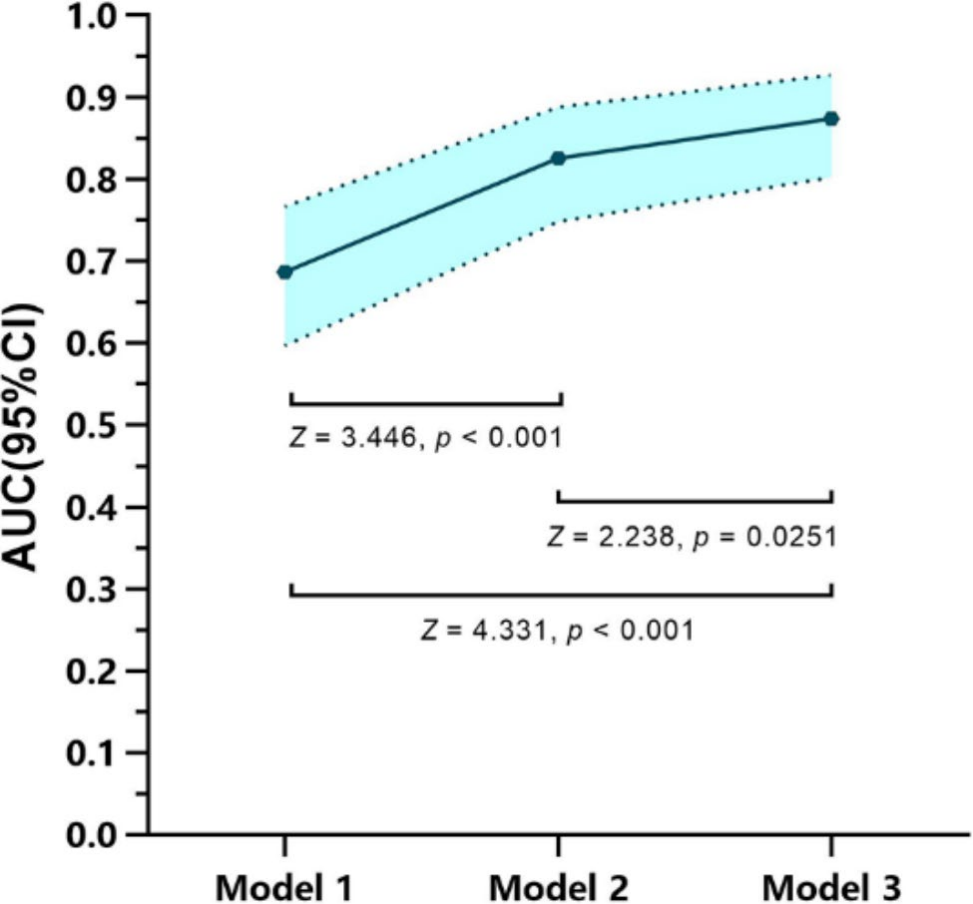
Pair-wise comparisons of AUC of the three models.The AUCs of mode 1, model 2 and model 3 were significantly different

**Fig. 9:**
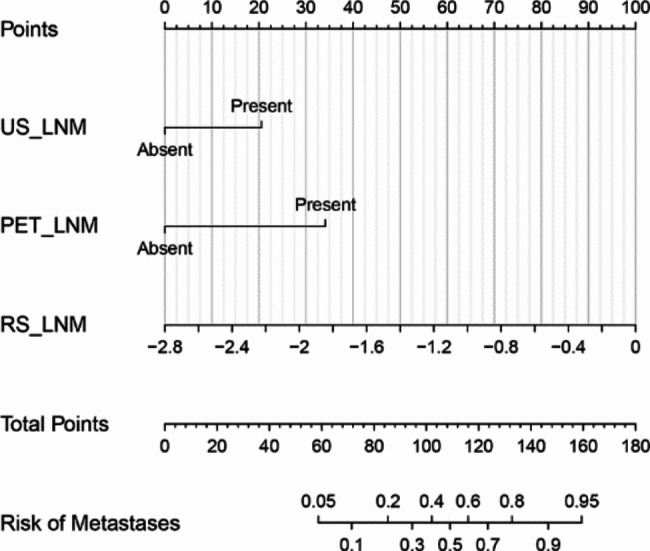
Nomogram for model 3

The results showed that T stage, US_LNM, and PET_LNM were associated with RS_LNM. Three models with multivariate were constructed for predicting LNM, as shown in Table 2. RS_LNM was an independent predictor when US_LNM and PET_LNM were integrated in the multivariable model, with OR value of 8.078 [95%CI, (1.862–35.050), *P* < 0.05]. The differentiation of the three models was shown in Table 3. The ROC curves showed that model 3 outperformed model 1 for the sensitivity (model 3 vs. model 1, 82.86% vs. 48.57%), and outperformed model 2 for the specificity (model 3 vs. model 2, 82.02% vs. 68.54%). The AUC of mode 1, model 2 and model 3 was 0.687, 0.826 and 0.874, seen as Fig. 7, and the Delong test showed the AUC of model 3 was significantly higher than that of model 1 and model 2, seen as Fig. 8. The nomogram was the visualization of the model 3, seen as Fig. 9.

**Table 2:** Multivariate analysis

Parameters	Model 1: T stage + US_LNM		Model 2: T stage + US_LNM + PET_LNM		Model 3: T stage + US_LNM + PET_LNM + RS_LNM	
	OR(95%CI)	*p*	OR(95%CI)	*p*	OR(95%CI)	*p*
T stage						
US_LNM	7.461(2.932–18.984)	< 0.001	4.007(1.407–11.411)	0.009	3.324(1.081–10.217)	0.036
PET_LNM			9.467(3.373–26.572)	< 0.001	7.349(2.518–21.448)	< 0.001
RS_LNM					8.078(1.862–35.050)	0.005

**Table 3:** Distinguishability of the three models

Parameters	AUC(95%CI)	*p*	Cut-off		Se(%)	Sp(%)	Corrected AUC
Model 1	0.687(0.597–0.767)	< 0.001		0.19	48.57	88.76	0.686
Model 2	0.826(0.748–0.888)	< 0.001		0.07	88.57	68.54	0.823
Model 3	0.874(0.802–0.927)	< 0.001		0.28	82.86	82.02	0.864

Note: Corrected AUC by Bootstrap1000

**Fig. 10:**
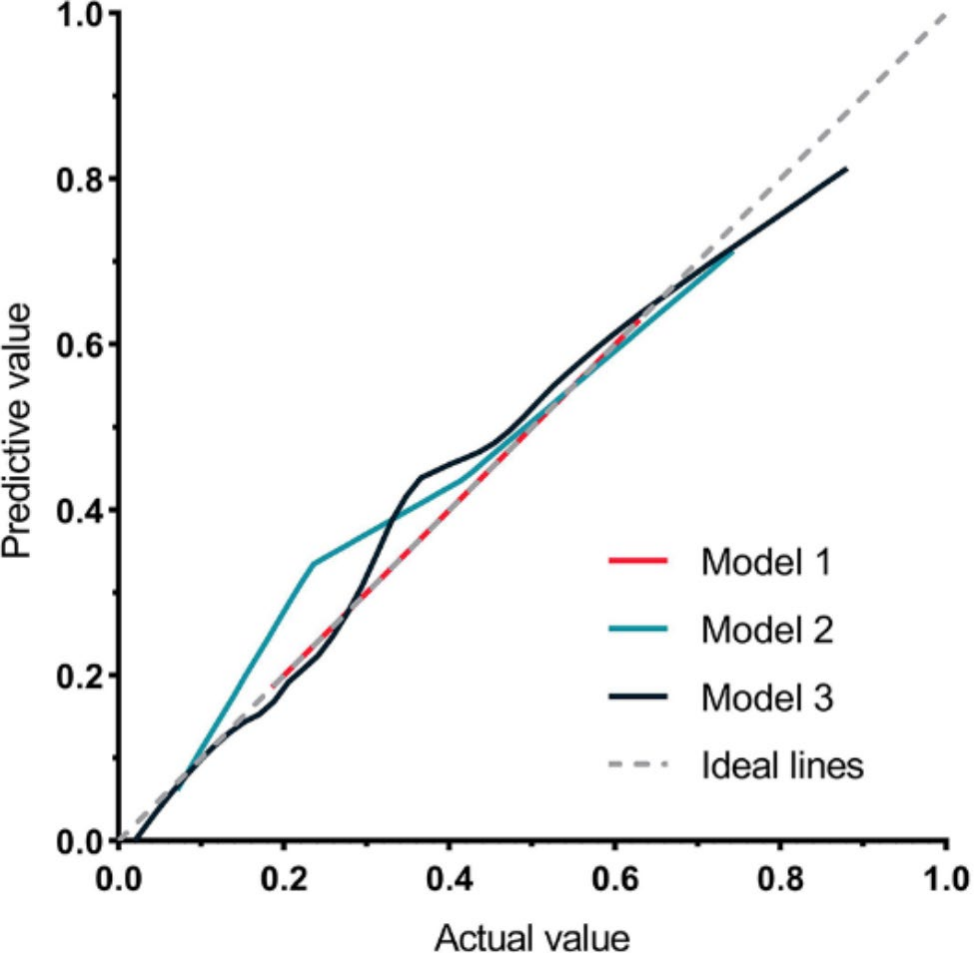
Calibration curves for the three models

The calibration curves for all three models showed good calibration, seen as Fig. 10. The continuous NRI for model 2 relative to model 1 was 1.118 (95% CI: 0.797–1.422), p < 0.001, and IDII of 0.141 (95% CI: 0.078–0.203), which mean positive improvement. The continuous NRI for model 3 relative to model 2 was 0.666 (95% CI: 0.368–0.963), p < 0.001, and IDII of 0.060 (95% CI: 0.007–0.114), p = 0.026, which mean improvement.

**Fig. 11:**
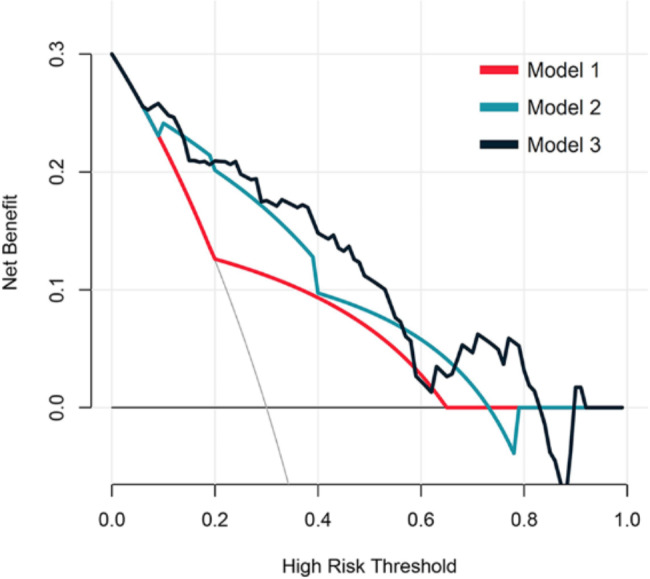
Decision curves of the three models

Decision curve analysis showed that model 2 resulted in a higher degree of net benefit for all the patients than model 1, and model 3 resulted in a higher degree of net benefit for all the patients than model 2, seen as Fig. 11.

## Discussion

A large number of clinical data have confirmed that the prognosis of breast cancer patients is closely related to the presence or absence of axillary lymph node metastasis [1, 3]. Traditional imaging methods commonly used to evaluate the metastatic status of axillary lymph nodes in breast cancer are mostly based on the subjective experience of imaging physicians, semi-quantitative or quantitative analysis of low dimensions, and a lot of deep and high-dimensional data information has not been fully exploited.There is an urgent need for noninvasive methods that can accurately assess axillary LNM in BC patients preoperatively, thereby reducing the need for anterior lymph node biopsy and axillary lymph node dissection. Such noninvasive methods are important for guiding the choice of axillary surgical modality and improving the quality of life of BC patients.

Recent studies have shown that radiomics have a good predictive power for evaluating LNM various cancers [9, 10]. Radiomics features are the product of genotypic and phenotypic influences of tissues that can reflect the biology of tumors [15] .The term “-omics” originated in molecular biology to characterize DNA, RNA, proteins, and metabolites [15]. In medical imaging research, radiomics is the analysis of images to obtain data that may be relevant to clinical outcomes and provide reliable potential imaging-based biomarkers for improving diagnosis, optimizing treatment plans, and predicting outcome [16, 17].Algorithm-based medical imaging features have the advantages of being non-invasive, sample-independent, real-time, and not limited to the tissues being examined compared to tissue-based biomarkers.The current approach of predicting axillary LNM in BC using radiomics evaluates the axillary lymph node images obtained by X-ray mammography, ultrasound, and MRI, of which evaluation of the ultrasound scans are the most frequently used for diagnosis. Mao et al. [18] predicted axillary LNM based on mammography radiomics with an AUC of 0.79; Qiu et al. [19] predicted axillary LNM based on breast ultrasound radiomics with an AUC of 0.759; and Tan et al. [20] predicted axillary LNM based on breast MRI radiomics with an AUC of 0.805. Lee et al. [21] and Gao et al. [22] achieved good predictive results based on breast ultrasound radiomics to evaluate axillary LNM.

Studies have demonstrated the potential application of PET radiomics in the diagnosis, staging, and assessment of treatment response in breast cancer [8]. The application of PET radiomics has not been widely studied in the diagnosis of BC LNM; however, it has shown to improve the diagnostic sensitivity for LNM patients with BC [23]. As a non-invasive, visual method that can quantify the entire tumor heterogeneity, PET radiomics can reflect the biological characteristics of tumors more objectively and comprehensively by extracting quantifiable image features from the ROI of PET images in high throughput, creating high-dimensional datasets, and mining the features associated with tumors through data mining analysis techniques [24]. In previous studies [22, 23], PET imaging-based histology of primary BC was analyzed to predict axillary lymph node status with AUCs of 0.64 and 0.89, respectively, thus showing a large difference in diagnostic efficacy. Therefore, in this study, a comprehensive model (model 3) was constructed to predict axillary LNM based on PET radiomics in addition to the evaluation of the clinical, pathological, ultrasound, and PET/CT parameters. The results showed that model 3 had higher a discrimination and calibration for predicting LNM in BC, with positive improvements in both continuous NRI and IDII, relative to the other two models. Model 3 had a stronger predictive performance as well as a net benefit for more patients.

Previous studies have often predicted LNM by the volume of the primary tumor and its metabolic parameters. For example, studies by De [25] and Song et al. [5, 26] showed that the metabolic activity of the primary tumor obtained by ^18^ F-FDG PET/CT in rectal, gastric, and BCs was positively correlated with LNM. In contrast, SUVmax, SUVmean, SD, and MTV did not significantly correlate with axillary LNM the present study. Another study [23] showed that data on pathological classification, molecular subtypes, and immunohistochemistry were not associated with axillary LNM, and the present study was similar to these results.

Limitations of this study are that it was a retrospective single-center study with possible selection bias; patients with multifocal lesions, bilateral lesions, and occult lesions were excluded because it was difficult to identify lesions that would lead to LNM; and only internal validation was performed due to the volume of data, which needs to be expanded for external validation.

## Conclusion

In this study, a comprehensive model to diagnose axillary LNM was constructed based on clinicopathology, ultrasound, PET/CT, and PET radiomics. This model with a high sensitivity (82.86%), specificity (82.02%), and an AUC of 0.874 can achieve a non-invasive, individualized, precise, and holistic presurgical assessment of axillary LNM in BC patients. Further controlled prospective studies are needed to validate the predictive accuracy of this comprehensive model.

## Data Availability

We confirm that all the materials and data with regard to the analysis in the manuscript are available for request. Please contact Yan Li for data request.

## Abbreviations

LNM: Lymph Node Metastasis
BC: Breast Cancer
^18^ F-FDG: ^18^ F-fluorodeoxyglucose
MTV: Metabolic Tumor Volume
ROI: Region of Interest
SUV: Standardized Uptake Value
LASSO: Least Absolute Shrinkage and Selection Operator
RS: Radiomics Score
AUC: Area Under the Curve
NRI: Net Reclassification Index
IDII: Integrated Discrimination Improvement Index

## Declarations

### Clinical trials

Date of first registration: 24/04/2023.

ClinicalTrials.gov Identifier: NCT05826197.
